# Interspecific Gene Exchange Introduces High Genetic Variability in Crop Pathogen

**DOI:** 10.1093/gbe/evz224

**Published:** 2019-10-11

**Authors:** Alice Feurtey, Danielle M Stevens, Wolfgang Stephan, Eva H Stukenbrock

**Affiliations:** 1 Environmental Genomics, Max Planck Institute for Evolutionary Biology, Plön, Germany; 2 Botanical Institute, Christian-Albrechts University of Kiel, Germany; 3 Department of Plant Pathology, University of California, Davis; 4 Leibniz Institute for Evolution and Biodiversity Science, Berlin, Germany

**Keywords:** interspecific hybridization, recurrent introgression, genome evolution, fungal pathogens

## Abstract

Genome analyses have revealed a profound role of hybridization and introgression in the evolution of many eukaryote lineages, including fungi. The impact of recurrent introgression on fungal evolution however remains elusive. Here, we analyzed signatures of introgression along the genome of the fungal wheat pathogen *Zymoseptoria tritici*. We applied a comparative population genomics approach, including genome data from five *Zymoseptoria* species, to characterize the distribution and composition of introgressed regions representing segments with an exceptional haplotype pattern. These regions are found throughout the genome, comprising 5% of the total genome and overlapping with > 1,000 predicted genes. We performed window-based phylogenetic analyses along the genome to distinguish regions which have a monophyletic or nonmonophyletic origin with *Z. tritici* sequences. A majority of nonmonophyletic windows overlap with the highly variable regions suggesting that these originate from introgression. We verified that incongruent gene genealogies do not result from incomplete lineage sorting by comparing the observed and expected length distribution of haplotype blocks resulting from incomplete lineage sorting. Although protein-coding genes are not enriched in these regions, we identify 18 that encode putative virulence determinants. Moreover, we find an enrichment of transposable elements in these regions implying that hybridization may contribute to the horizontal spread of transposable elements. We detected a similar pattern in the closely related species *Zymoseptoria ardabiliae*, suggesting that hybridization is widespread among these closely related grass pathogens. Overall, our results demonstrate a significant impact of recurrent hybridization on overall genome evolution of this important wheat pathogen.

## Introduction

Previous studies of fungal pathogen genomes have revealed exceptionally high levels of genomic variability (Möller and Stukenbrock 2017). Some of this variation is considered adaptive, allowing pathogens to cope with variation in host immune genes and overcome immune responses leading to infection in plant host tissues. Rapid evolution in fungal pathogen genomes has been associated with specific, repeat-rich genome compartments including accessory chromosomes, gene clusters, and repeat islands (Raffaele and Kamoun 2012; Möller and Stukenbrock 2017). In a few examples, interspecific hybridization has also been demonstrated as a driver of rapid evolution (e.g., Brasier and Kirk 2010; Inderbitzin et al. 2011; Leroy et al. 2016). In fungi, hybridization can occur by vegetative fusion of hyphae or by recombination between nonconspecific individuals (Schardl and Craven 2003; Feurtey and Stukenbrock 2018).

Analyses of genome data have revealed new insights on how hybridization can provide a mechanism for the emergence of new plant pathogens and novel host specificities within few years (Stukenbrock, Christiansen, et al. 2012; Menardo et al. 2016; Depotter et al. 2018). Hybridization gave rise to a new mildew pathogen on the crop species triticale, a hybrid cereal that was introduced in the 1960s (Menardo et al. 2016). This crop was initially resistant to the powdery mildew pathogen *Blumeria graminis*; however, a new virulent form of the pathogen, able to overcome this resistance, has emerged in Europe in the last decade (Walker et al. 2011; Troch et al. 2012; Menardo et al. 2016). Comparative population genomic analyses including data from several *B. graminis* formae speciales revealed that the genome of *B. graminis* f. sp. *triticale* comprises a mosaic structure with genomic segments of different origin and with high similarity to either the wheat infecting mildew form *B. graminis* f. sp. *tritici* or the rye infecting form *B. graminis* f. sp. *secalis*. The distribution of genomic segments in *B. graminis* f. sp. *triticale* is consistent with a recent hybrid origin of the triticale infecting form by sexual mating between *B. graminis* f. sp. *tritici* and *B. graminis* f. sp. *secalis* (Menardo et al. 2016). The study provides a prominent example of how new pathogens of crops can evolve by hybridization, and it demonstrates how the history of hybridization events can be recovered from genome data. Introgressive hybridization was also shown to contribute to the genome evolution of other important fungal crop pathogens including *Verticillium longisporum* causing stem striping disease on oilseed rape and the apple scab pathogen *Venturia inaequalis* (Leroy et al. 2016; Depotter et al. 2017).

A population genomic study of a fungal grass pathogen, *Zymoseptoria pseudotritici*, provided detailed insight into the population genetics and demography associated with hybridization (Stukenbrock et al. 2012). In this particular case, the hybrid lineage emerged from a single sexual cross between two parental individuals. Consequently, the genome of *Z. pseudotritici* is almost deprived of variation: the genome alignment is a mosaic of segments representing either one or two haplotypes from the two parental individuals. Additional polymorphisms have not been introgressed from any of the parental species, and the only source of new variation is spontaneous mutations that accumulate at low frequency along the genome. The parental haplotypes exhibit on an average 3% divergence indicating that hybridization has occurred between two closely related, but otherwise isolated, species (Stukenbrock et al. 2012). *Zymoseptoriapseudotritici* has so far only been isolated from wild grasses in Iran (Stukenbrock et al. 2012). The biology of the species is poorly understood, and the parental species of the *Z. pseudotritici* hybrid is not known. However, *Z. pseudotritici* co-occurs with other *Zymoseptoria* species infecting other wild grasses, including *Zymoseptoriaardabiliae* and *Zymoseptoriabrevis* (Quaedvlieg et al. 2011; Stukenbrock et al. 2011). The unique hybridization event that gave rise to *Z. pseudotritici* may have involved parental species infecting distinct hosts and possibly isolated by their distinct host specificities.

*Zymoseptoria* *pseudotritici* is one of the closest relatives of the prominent wheat pathogen, *Zymoseptoria tritici*, the causal agent of the disease Septoria tritici blotch. The two species shared a common ancestor ∼12,000 years ago (Stukenbrock et al. 2006, 2011). In contrast to *Z. pseudotritici*, the genome of *Z. tritici* is characterized by high levels of genetic variation, including substantial structural variation in its core chromosomes and a set of highly variable accessory chromosomes (Goodwin et al. 2011; Croll et al. 2013; Plissonneau et al. 2016). Moreover, the nucleotide diversity along the 40-Mb haploid genome is remarkably high; a recent study identified 1.4 million SNPs corresponding to a Watterson’s theta value of 0.014 (Stukenbrock and Dutheil 2018). Mechanisms that contribute to this high variation in *Z. tritici* involve exceptionally high rates of recombination (Croll et al. 2015; Stukenbrock and Dutheil 2018) and prominent activity of transposable elements (TEs) that mediate structural variation along the genome (Plissonneau et al. 2016). Moreover, one study reported signatures of introgression in spliceosomal intron regions through detailed analyses of the distribution of selfish group II introns in *Z. tritici*, *Z. pseudotritici*, and *Z. ardabiliae* (Wu et al. 2017).

In the present study, we have carefully analyzed genomic regions in *Z. tritici* with exceptionally high levels of genetic variation. We demonstrate how these regions cannot be recovered in population genomic data based on reads mapping but only in genome alignments generated from de novo genome assemblies. We also show that these “outlier” regions show genomic signatures compatible with interspecific gene flow with other *Zymoseptoria* species. We extended our analyses to include the other sister species *Z. ardabiliae* and confirm that these genomic signatures are a common occurrence among this group of fungi.

## Materials and Methods

### Generation of Multiple Genome Alignments

To assess the distribution of intra- and interspecific genetic variation along the 40-Mb haploid genome of *Z. tritici* and its sister species, we generated four whole-genome alignments. The four genome alignments were based on de novo genome assemblies of either Illumina short read data or PacBio SMRT long read data (see supplementary fig. S1 and table S1, Supplementary Material online).

For the first multiple genome alignment (hereafter “All-Zt MGA”), we used already published population genome sequencing data for 25 *Z. tritici* isolates, including two genomes sequenced with PacBio technology (Stukenbrock et al. 2011; Mcdonald et al. 2017; Haueisen et al. 2019). As a backbone for the alignment, we included the Sanger-sequenced reference genome of *Z. tritici* isolate IPO323, which includes 21 fully assembled chromosomes (Goodwin et al. 2011). Furthermore, we included one genome each of Z. *ardabiliae*, *Z. brevis*, and *Z. pseudotritici* sequenced with PacBio technology and one genome of *Zymoseptoriapasserinii* sequenced with Illumina technology (Stukenbrock et al. 2011).

For the second multiple genome alignment (hereafter “All-Za MGA”), we used Illumina resequencing data from 17 *Z. ardabiliae* isolates (Stukenbrock and Dutheil 2018). Additionally, we included the reference genome of *Z. tritici* IPO323, one Illumina assembly of *Z. passerinii* and the PacBio assemblies of *Z. brevis*, *Z. pseudotritici*, and *Z. ardabiliae*.

Finally, two genome alignments were generated to validate regions of high variability in the *Z. tritici* genome. One alignment (hereafter “3-Zt PacBio MGA”) included three high-quality assembled genomes of *Z. tritici*: the Sanger-sequenced reference genome of IPO323 and the PacBio assemblies of the isolates Zt05 and Zt10. The other alignment (“3-Zt Illumina MGA”) included the IPO323 reference sequence and the Illumina assemblies of Zt10 and Zt05.

The pipeline used to create and process the four multiple genome alignments was adapted from (Stukenbrock and Dutheil 2018). Details and alignment filters can be found in the supplementary text and figures S1–S3, Supplementary Material online. To compare coordinate positions between alignments, all alignments were projected against the reference genome of IPO323. Alignment statistics are summarized in supplementary table S2, Supplementary Material online.

### Variant Calling from Multiple Genome Alignments and Reference-Based Mapping

Based on the filtered multiple genome alignments, we extracted variable sites using the option “VcfOutput” in MafFilter to obtain a list of variable positions in vcf format (Dutheil et al. 2014).

We further compared the content and quality of the SNP data set produced by alignment of de novo assembled genomes of *Z. tritici* isolates (3-Zt PacBio MGA) to a SNP data set obtained by a read mapping approach of the same isolates. In brief, we aligned the Illumina reads of isolates Zt05 and Zt10 to the IPO323 reference with the program bwa v.0.7.15 using the mem algorithm (Li and Durbin 2009) and called variants with the program GATK (McKenna et al. 2010; DePristo et al. 2011). Details about data filtering and processing are summarized in the supplementary text, Supplementary Material online. To compare vcf files obtained from the multiple genome alignments and the reference-based assemblies, we used custom made scripts in R and Python. Scripts and data used in this article can be found on Zenodo (doi: 10.5281/zenodo.3377936).

### Diversity Analyses and Introgression Detection

Based on the multiple genome alignments, we inferred the distribution of intra- and interspecific genetic variation along the *Zymoseptoria* genomes. We used MafFilter (option “DiversityStatistics”) to compute the number of segregating sites, Theta, Pi, and Tajima’s *D* per window (Dutheil et al. 2014).

The All-Zt MGA and All-Za MGA were first divided into 1 kb sliding windows (slide = 500 bp) and windows smaller than 500 bp were removed. We also filtered out sequences in each alignment block that was shorter than 80% of the entire block length. Potential local gene flow was first assessed by generating phylogenetic trees in windows along the genome using MafFilter with the maximum likelihood estimate of distance and the BioNJ method for reconstructing the tree (Dutheil et al. 2014). Signatures of introgression were recognized as windows in which the *Z. tritici* sequences are nonmonophyletic using the ete3 python library (Huerta-Cepas et al. 2016). For this analysis, only windows in which all species were represented were included.

We further validated the signatures of introgression in the *Z. tritici* genome by computing the parameter Gmin along the All-Zt MGA and All-Za MGA (Geneva et al. 2015). For each window, we generated a distance matrix between sequences using the “identity” DistanceCalculator of the BioPython library. For *Z. ardabiliae*, *Z. pseudotritici*, and *Z. brevis*, we divided the minimum distance between any *Z. tritici* isolate and the sister species sequences by the mean of the distances between sequences of the two species.

Finally, the R package regioneR (Gel et al. 2016) was used to fuse neighboring windows with a signature of introgression or with high variability (highly variable regions defined by 1 kb windows with >200 segregating per window) to call the coordinates of these loci.

### PCR-Based Validation of Highly Variable Genomes in the *Z. tritici* Genome

In order to validate the existence of the highly variable regions (HVRs), we amplified some of these regions using a PCR assay designed to bridge between conserved and HVRs. For extraction of DNA, *Z. tritici* isolates (*N* = 13) were maintained on solid YMS agar (0.4% [w/v] yeast extract, 0.4% [w/v] malt extract, 0.4% [w/v] sucrose, 2% [w/v] bacto agar) (Haueisen et al. 2019). Fungal material was lysed using glass beads and 500 µl of lysis buffer. Then 500 µl of a 1:1 phenol/chloroform mix was added and the tubes were shaken for 30 min. The supernatant was extracted and the DNA was precipitated using ethanol. Finally, the DNA was purified using RNase. The sequences of primers specifically designed for this study are listed in supplementary table S3, Supplementary Material online. The reaction mixture for PCRs was as follows: 1× Phusion High-Fidelity Polymerase Master Mix (Invitrogen, Carlsbad, CA), 10 µM of each primer, 9–100 ng of genomic DNA template, and double distilled water to a final volume of 10 µl. PCR conditions were 98 °C for 30 s; 35 cycles of 98 °C for 5 s, 63 °C for 20 s, and 72 °C for 30 s; 72 °C for 10 min. The annealing temperature for each primer pair was optimized through gradient PCR. Extension times were adjusted to ∼30 s per kilobase of DNA amplified. Amplicons were visualized on 0.8% TAE agarose gel containing SYBR Safe DNA gel stain (Invitrogen, Carlsbad, CA).

### Functional Predictions

We used the gene and TE annotations from the *Z. tritici* IPO323 reference genome (Grandaubert et al. 2015) to correlate signatures of introgression to genome features including genes and TEs. To this end, we used the R package regioneR to test the overlap between HVRs, predicted gene positions and TEs (Gel et al. 2016). This package implements a randomization strategy to statistically assess the association between sets of genomic regions by replacing one of the sets randomly along the genome. We used the permutation function “randomizeRegions” and 1,000 permutations. Two limiting conditions were applied for the randomization: 1) As the ratio, we used to detect introgression is only powerful enough in regions of the genome with a sufficient amount of information, we masked all regions with <12 *Z. tritici* sequences. This threshold of 12 corresponds to the minimum number of sequences in the windows with a signature of introgression as identified by the phylogenetic approach as well as by the Gmin-based approach. 2) The randomization was done per chromosome whereby regions from chromosome 1 were randomly placed on chromosome 1 and not on any other chromosome.

The software SnpEff version 4 was used to predict the effect of the variants detected in the previous steps on predicted protein sequences (Cingolani et al. 2012). Such effects are classified on a scale that ranges from “low” impact variants (e.g., synonymous mutations) to “high” impact variants that can change the reading frame, add stop codons or modify split sites. The whole list of effects considered as “high,” “moderate,” and “low” impact can be found in the SnpEff manual (Cingolani et al. 2012). We used the output of this analysis to assess if “high” impact SNPs are enriched in the introgressed regions. Furthermore, we assessed if effector candidates (Stukenbrock and Dutheil 2018) were enriched in the introgressed regions using the “resampleRegions” from regioneR (Gel et al. 2016).

## Results

### The Genome of *Z. tritici* Contains HVRs

**Fig. 1.**
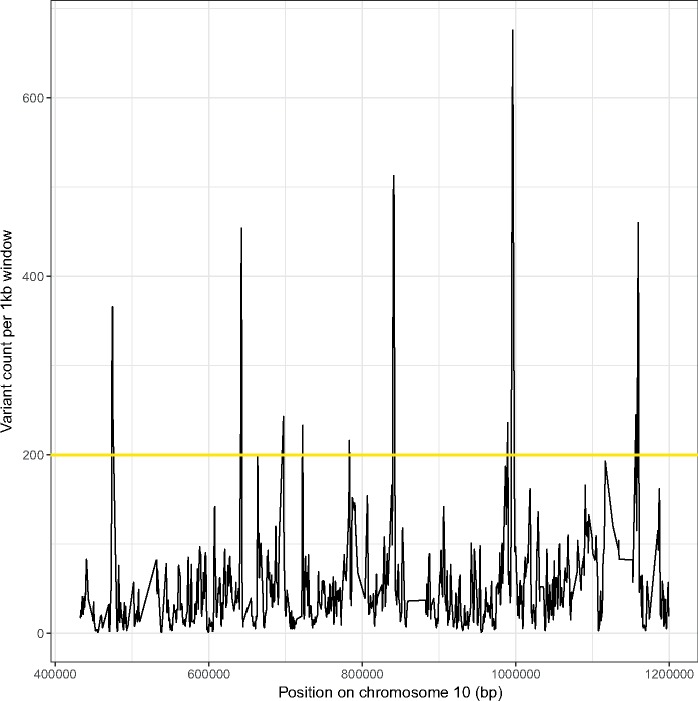
—Highly heterogeneous distribution of genetic variation along the genome of *Zymoseptoria tritici*. Number of variant positions in 1 kb window along a randomly selected region in chromosome 10 as computed from a multigenome alignment of 26 *Z. tritici* genomes.

We first generated a full genome alignment based on de novo assemblies of 26 genomes of *Z. tritici* and high-quality genome assemblies of *Z. brevis*, *Z. pseudotritici*, *Z. ardabiliae*, and *Z. passerinii* (All-Zt MGA). This alignment was used as input for the software MafFilter (Dutheil et al. 2014) to compute the number of variable sites along the *Zymoseptoria* genome. We inferred a total of 6,520,454 variable sites in the 27-Mb alignment comprising all genomes. The intraspecific variation in *Z. tritici* corresponds to 1,315,411 variable sites. We analyzed the distribution of intraspecific variation in *Z. tritici* along the All-Zt MGA genome alignment in sliding windows of 1 kb (slide window size 500 bp). The distribution of polymorphisms showed a highly heterogeneous pattern along the chromosomes with unexpectedly high peaks of variation contained within short regions (fig. 1). The filtered alignment with a length of ∼26 Mb (supplementary table S2, Supplementary Material online) comprises 51,539 1-kb windows with sites segregating in *Z. tritici*, and of these windows, 1,805 have >200 segregating sites. We defined a threshold to distinguish windows with >200 segregating sites within 1 kb windows as highly variable windows, and we joined consecutive windows in this category to have a map of HVRs along the *Z. tritici* genome. While these windows in total comprise a small proportion, 3.5% of the total alignment (i.e., 665 HVRs with a total length of 990,074 bp), they contain a considerable amount of the variation, 24% of the total number of segregating sites (in HVRs: 315,060 and in the total alignment: 1,315,411).

As a measure of allelic frequencies, we computed Tajima’s *D* in the HVRs and the remaining genome. Interestingly, we find that Tajima’s *D* is considerably higher in windows located in the HVRs: the median Tajima’s *D* value in windows of HVRs is 1.0, while it is −0.04 in windows of the remaining genome. Tajima’s *D* is positive when there is an excess of variants of intermediate frequencies. The higher Tajima’s *D* values in the HVR windows thereby show that these regions comprise a higher abundance of variants that are shared by different individuals. Manual inspection of the several HVRs confirmed this and revealed a pattern of two or more highly diverged haplotype groups within these regions (see supplementary fig. S4*A*–*C*, Supplementary Material online, for an exemplary visualization of the haplotype pattern in the alignment).

### Validation of the HVRs

The unexpected pattern of genomic variation in *Z. tritici* may arise either from technical bias in the processing of genome data or from actual demographic events in populations of *Z. tritici*. To validate that the high amount of variation in the alignment did not originate from assembly errors, we used two *Z. tritici* strains, Zt05, and Zt10, for which both Illumina and PacBio genome data are available. We included the independently generated assemblies, one generated from paired-end Illumina reads and the other from long SMRT reads, of the same isolates in two separate alignments: 3-Zt Illumina and 3-Zt PacBio. Longer reads are expected to facilitate de novo genome assemblies, therefore, if the observed high local variation is due to assembly errors, we expect more variation in the 3-Zt Illumina alignment compared with the 3-Zt PacBio alignment. However, we find that the numbers of segregating sites in the two genome alignments are very similar: 1,048,538 SNPs in 3-Zt Illumina and 1,067,522 SNPs in 3-Zt PacBio with a consistent distribution along the genome, including in the windows with high variability (supplementary fig. S4*A*, Supplementary Material online). In total, we compared SNP counts in 60,913 1-kb windows with data for both alignments and found that only 6.5% (3,944) of the compared windows showed different numbers of SNPs. In the Illumina-based alignment, 56,969 windows had SNP counts comparable (in a 10% range) to the corresponding window in the PacBio-based alignment, leaving 3,944 (6.5%) windows with a different SNP count. Among these, 70% had a difference of <16 segregating sites. From this, we conclude that assembly artifacts do not explain the high local variation observed.

To experimentally validate the presence of HVRs and the observed haplotype patterns, we used PCR to amplify specific haplotypes with pairs of primers bridging the transition between HVR and the neighboring loci: one primer was designed in a conserved region and several others in the HVRs allowing us to amplify selectively the different haplotypes. Such primer design generated an “amplification/nonamplification” matrix that we compared with the distribution of haplotypes that we expect based on the multiple genome alignments (supplementary table S4, Supplementary Material online). We validated the amplification pattern of 11 loci for 13 samples and with some exceptions (4 unpredicted amplifications over 429 reactions) confirmed the haplotype distribution and sequence variation predicted from the multiple genome alignment.

Finally, we used a reference-based mapping approach to compare the distribution of SNPs. Interestingly, using this approach, we were not able to recover the majority of the HVRs. Indeed, the windows in the HVRs detected in the genome alignment correspond to regions of either low or no variability in the reference-based SNP data set (supplementary fig. S4*B*, Supplementary Material online). We compared the genotypes resulting from both variant-calling methods at each position to determine whether the differences in variants came from different genotypes being called, that is, erroneous alleles at these positions, or missing data. Of the 762,543 total sites found to be variable, 378,431 sites were identified as being variable by both approaches, 308,908 were found in the multigenome alignment only, and 75,204 in the reference-based mapping only. Among the differently identified SNPs, we found that these differences were due to missing data in >95% of the cases (*N* = 371,731). We conclude that only the alignment of de novo assembled genomes allows for the detection of such highly variable genome segments as the HVRs identified in the genome of *Z. tritici*.

In summary, two independent genome alignments and a PCR assay confirmed the existence of HVRs in the genome of *Z. tritici*. However, we show that these regions cannot be recovered by mapping of Illumina reads to a reference genome due to the exceptionally high local sequence divergence in these regions.

### Signatures of Introgression Overlap with HVRs in the *Z. tritici* Genome

We next addressed the origin of the HVRs and the distinct haplotype patterns observed in these regions. Based on the sequence variation, we hypothesized that these regions reflect introgression in the *Z. tritici* genome. To test this hypothesis, we used the All-Zt MGA, which contains sequences from five different *Zymoseptoria* species (*Z. tritici*, *Z. pseudotritici*, *Z. brevis*, *Z. ardabiliae*, and *Z. passerinii*), to assess potential traces of introgression among these closely related species. First, we created a phylogenetic tree for every 1 kb window along the genome using MafFilter (Dutheil et al. 2014). For each tree, we assessed whether the *Z. tritici* isolates form a monophyletic group or if the *Z. tritici* isolates were nonmonophyletic with *Z. tritici* sequences clustering with sequences of the sister species. We applied strict filtering criteria to ensure the absence of confounding effects such as alignment errors in the HVRs (see Materials and Methods) and thereby extracted phylogenetic trees from 18 Mb of high-quality genome alignment. We classified windows as being “nonmonophyletic” or “monophyletic” based on the clustering of *Z. tritici* sequences in the trees. We considered introgression to be reflected in windows were *Z. tritici* represents a nonmonophyletic group. Indeed, we find 3,532 nonmonophyletic regions along the *Z. tritici* genome in comparison to 31,603 monophyletic windows. To compute the length distribution of nonmonophyletic segments, we joined consecutive windows with the same tree topology; in total, the nonmonophyletic windows includes 1,903 regions comprising of 2,425,801 bp. The mean length of these segments is 1,275 bp and the maximum length is 10,230 bp. In contrast, the monophyletic segments cover a total of 15,757,555 bp with a mean length of 2,831 bp and a maximum length of 26,756 bp. We consider that the variation in lengths of segments reflects different ages of potential introgression events. In a sexually recombining organism such as *Z. tritici*, recombination breaks down the linkage of haplotype blocks as time increases. Thereby recurrent hybridization events followed by multiple generations of backcrossing leave introgressed segments of varying size in the genome of *Z. tritici* where long segments represent young events.

**Fig. 2.**
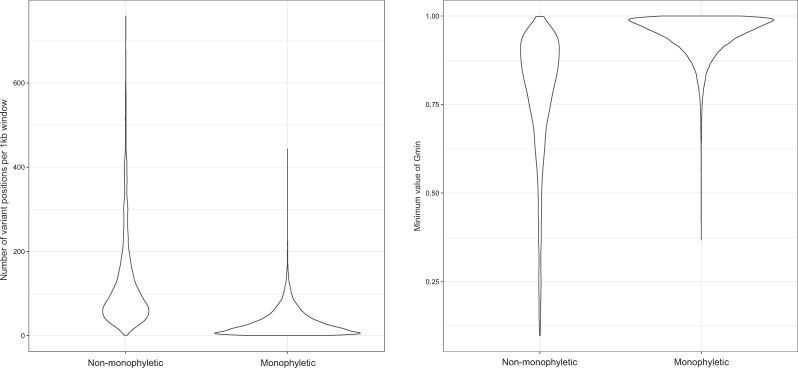
—Distribution of variable sites in monophyletic and nonmonophyletic 1 kb windows in the genome of *Zymoseptoria tritici*. Violin plots showing the number of variant sites for monophyletic and nonmonophyletic windows (left) and the distribution of minimum Gmin values obtained from a comparison of *Z. tritici* and each of the other *Zymoseptoria* species in monophyletic and nonmonophyletic windows (right).

To further test the hypothesis of introgression in the *Z. tritici* genome, we compared the overlap of nonmonophyletic windows and the distribution of values of another measure of divergence, Gmin. We computed the parameter Gmin as a measure of sequence differentiation within and between species and defined windows with a clear signature of introgression as windows where the *Z. tritici* isolates are nonmonophyletic and where the value of Gmin is <0.85 in one of the pairwise analyses of *Z. tritici* with the other *Zymosetoria* species (fig. 2). We fused windows that exhibited both signatures of introgression resulting in 1,129 segments with a clear signal of introgression comprising 1,398,089 bp (mean length = 1,238 bp, max length = 10 kb).

Next, we correlated the distribution and frequency of introgression signals with coordinates of the HVRs. Most of the windows in the HVRs had a signal of introgression as indicated by the phylogenetic analysis and the Gmin estimates. Among the windows for which we could estimate a tree, only 86 (representing 13% of the highly variable windows) exhibited no signal of introgression (either based on the topology of phylogenetic trees or the Gmin value), whereas 460 exhibited both signatures of introgression (representing 77% of the highly variable windows). Likewise, the diversity (here estimated from the number of segregating sites per 1 kb window) is higher in windows where the *Z. tritici* isolates are nonmonophyletic (fig. 2). The median number of segregating sites in a monophyletic 1 kb window of *Z. tritici* is 22, while it is 85 in nonmonophyletic windows. Finally, we assessed the robustness of this finding by a permutation test assuming neutrality of the introgressed sequences. The overlap between the HVRs and the regions with introgression signals is significantly and strikingly more extensive than expected at random (width = 333,229 bp, *P* < 0.01). Based on the overlap of HVRs and segments with a strong signature of introgression, we conclude that the distinct patterns of nucleotide variation in the HVRs are consistent with a scenario of recurrent hybridization between *Zymoseptoria* species.

### Highly Variable Regions in *Z. tritici* Likely Originate from Introgression and Not Incomplete Lineage Sorting

Signatures of low divergence between closely related species can result from gene flow. However, other processes can also produce patterns of shared variation between species. An alternative explanation for the presence of shared variation between species is the retention of polymorphism from the common ancestor of the considered species, also termed incomplete lineage sorting (ILS). A substantial amount of ILS has been reported in *Z. tritici* previously (Stukenbrock et al. 2011), and we, therefore, set out to investigate if the HVRs could be the product of ILS rather than interspecific hybridization. The two processes can be distinguished by analysis of the length of haplotype segments (Racimo et al. 2015). Hybridization that has occurred after the split of the species as opposed to smaller segments of shared polymorphisms resulting from ILS. We used the equation presented in Racimo et al. (Racimo et al. 2015) to measure the expected length of shared haplotypes and the probability to observe fragments of a certain length. A recombination rate of 46 cM/Mb was estimated in the common ancestor of *Z. tritici*, *Z. pseudotritici*, and *Z. ardabiliae* from whole genome coalescence analyses (Stukenbrock et al. 2011), a value that is consistent (although slightly lower, making our estimations more conservative) with values obtained from experimental crosses of *Z. tritici* (Croll et al. 2015). The time of divergence between the lineages *Z. tritici* and *Z. ardabiliae* has previously been estimated to be 22,300 generations and between *Z. tritici* and *Z. pseudotritici* 11,000 generations (Stukenbrock et al. 2011). Using these values, we expect a length of 198 bp for shared haplotypes resulting from ILS between *Z. pseudotritici* and *Z. tritici* and of 97 bp for shared haplotypes between *Z. ardabiliae* and *Z. tritici*. We next compared the observed lengths of the HVRs to these haplotype lengths expected from ILS. The minimum threshold for the window size that we used to scan the genomes for signatures of introgression is already larger than the predicted length of haplotype fragments with introgression. Thus our analyses would not be able to detect regions small enough to be similar in size to these expectations under ILS. The average size of the haplotypes identified as outliers by our phylogenetic analysis and the Gmin parameter is 1,238 bp. The probability of retaining haplotypes of such length with an ILS assumption is 0.002 when using the estimated split time between *Z. pseudotritici* and *Z. tritici* and smaller than 0.001 for the estimated split time between *Z. ardabiliae* and *Z. tritici*. Although we cannot exclude that a fraction of the observed genomic variation is due to ILS, our analysis suggests that interspecific gene flow explains most of the variation detected in the HVRs.

### Regions Exhibiting Introgression Signatures Are Not Enriched with Genes Encoding Virulence Determinants

Next, we asked whether the wide distribution of introgression in the genome of *Z. tritici* could be functionally relevant. We, therefore, searched for overlapping windows with a signal of introgression and different annotated genomic features including TEs and protein-coding genes. Among these, we specifically considered genes predicted to be involved in plant–pathogen interaction, so-called effectors. With our strict filtering, we only included the genomic regions where the alignment had no missing data for any of the sister species used in the analyses. Hence, for this analysis, we only included the most conserved regions in the *Zymoseptoria* genome. As genomic regions enriched with TEs are typically more difficult to assemble and align, these are also among the alignment blocks that are most frequently excluded by our filtering approach. Therefore, the number of TEs was limited to 67 TEs in *Z. tritici*. Of these, 14 TEs overlap with the introgressed regions. Based on random permutations, we find that this is a significantly higher number than the number expected from a random distribution (*P* value <0.01) suggesting that TEs in the genomic regions affected by introgression. We note that we may underestimate the association of TEs and introgressed regions due to the strict data filtering.

**Fig. 3.**
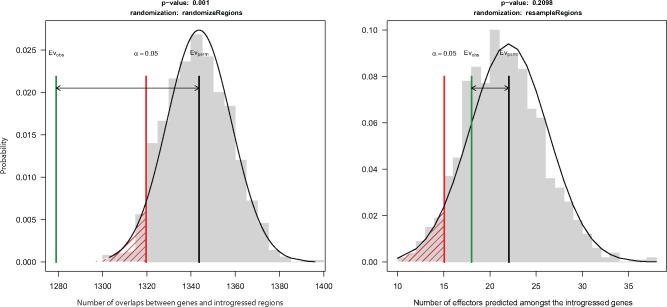
—Permutation test assessing possible associations in regions of the *Zymoseptoria tritici* genome showing signatures of introgression. On the left, association with predicted genes. On the right, association with predicted effectors among the genes overlapping with signatures of introgression. In gray, the random distribution obtained from 1,000 randomizations. The black line represents the median random value, the green line the observed value and the red line the threshold of significance (here, set at 0.05).

We found 1,279 genes (of 13,847 predicted gene models; Grandaubert et al. 2015) that overlap with windows with introgression signals. This number is lower than expected from random permutations (*P* value <0.01, fig. 3). Furthermore, we investigated the predicted effect on the protein sequences of the segregating sites detected in regions with a signature of introgression using SnpEff (Cingolani et al. 2012). Across the 1,279 genes overlapping with regions with a signature of introgression, 487 genes contained variants, which were classified as “high effect” mutations (including genes with the gain of a stop codon or the loss of a start codon), which is significantly more than expected at random (*P* value <0.01). This may indicate that more rapidly evolving genes are located in the genome regions affected by introgression.

To investigate a possible functional role of introgression, we searched for the enrichment of gene ontology (GO) categories using a previously published GO annotation (Grandaubert et al. 2015). The results, presented in supplementary table S5, Supplementary Material online, show enrichment of functions related to general cellular processes such as protein and DNA binding functions, but no enrichment of genes known to be associated with pathogenicity. To further address if introgression has influenced loci potentially involved in pathogenicity, we focused on genes encoding putative effectors. Using a previously published list of effectors genes (Stukenbrock and Dutheil 2018), we find there is no significant enrichment (*P* value = 0.2) of effectors in the introgressed regions although a small number (*N* = 18) colocalize with HVRs.

### Frequent Hybridization in the *Zymoseptoria* Genus

Finally, to identify which species potentially have a hybridization history with *Z. tritici*, we used the minimum Gmin value in the pairwise comparisons of *Z. tritici* with *Z. ardabiliae*, *Z. pseudotritici*, *Z. brevis*, and *Z. passerinii*. For each window previously identified to exhibit a signal of introgression, we identified the species with a Gmin value < 0.85. In some windows more than one species comparison showed a Gmin value < 0.85 and we were not able to identify a single source species: 32% of the HVR windows exhibited two species combinations with low Gmin values, 14% exhibited three species combinations with low Gmin values, and 5% four species combinations with low Gmin values. 47.8% of the windows in which the introgression signal was linked to a single species exhibited strong identify between *Z. tritici* and *Z. pseudotritici*. In 44.6% of these windows, the low Gmin was attributed to *Z. brevis*, and in 7.2% and 0.4% to *Z. ardabiliae* and *Z. passerinii*, respectively. This pattern correlates with the phylogenetic relatedness of *Z. tritici* to *Z. pseudotritici* and *Z. brevis* and may suggest that successful interspecific hybridization occurs more readily and more often between the more closely related species.

Knowing that several of the *Zymoseptoria* species infecting wild grasses were detected as source species of introgressed sequences, we hypothesized that introgression would not be limited to this species but that the signatures of hybridization also would be visible in population genomic data of other *Zymoseptoria* species. To test this, we used an alignment comprising 17 *Z. ardabiliae* genomes (All-Za MGA) to look for traces of introgression in this species using the same methods as described earlier. We identified 68 regions with introgression signals in *Z. ardabiliae* corresponding to 92,241 bp (supplementary table S2, Supplementary Material online). Comparing the genome-wide coordinates of introgressed regions, we find that 50,526 bp overlap with coordinates of introgressed windows in *Z. tritici*. 128 nonmonophyletic regions in the *Z. ardabiliae* alignment have an overlap with the identified introgressed regions in *Z. tritici* (80,379 bp out of 154,168 bp of the *Z. ardabiliae* genome). In summary, by conducting the same analyses using a population genomic data set of *Z. ardabiliae*, we find similar evidence of introgression as observed in *Z. tritici* suggesting that hybridization is a widespread phenomenon in this group of fungi.

## Discussion

Genome analyses of fungi reveal that interspecific hybridization occurs more frequently than previously thought. Hybridization was shown to promote the rapid evolution of fungal pathogens and in some cases, the emergence of new species (Brasier and Kirk 2010; Stukenbrock et al. 2012; Menardo et al. 2016). Population genomic analyses of the wheat pathogen *Z. tritici* have documented extensive genomic variability as a result of transposon activity and chromosome instability (Plissonneau et al. 2016; Möller et al. 2018). However, so far, hybridization has not been considered a relevant driver of genome evolution in this species despite recent reports of introgression in spliceosomal intron sequences (Wu et al. 2017).

In the present study, we investigated the genome-wide occurrence of genomic regions of exceptionally high variability in *Z. tritici* with the hypothesis that these originate from introgression. When analyzing these regions in 1 kb windows, we detect > 200 segregating sites between different isolates of *Z. tritici*. Because this extent of local within-species diversity is highly unusual, we first investigated the possibility of artifacts resulting from data handling or analyses. One possible artifact could be the unintended alignment of distinct paralogs rather than alignment of orthologous sequences. However, by assembling genomes de novo and careful filtering of whole-genome alignments generated from syntenic chromosome fragments, we consider it unlikely that the unusual variation represents the alignment of distinct paralogs. Second, the high variability could originate from misassemblies of short Illumina reads. To address this possible artifact, we used Illumina and PacBio resequencing data of the *Z. tritici* strains Zt05 and Zt10. The PacBio technology produces considerably longer reads than Illumina sequencing, which facilitates the de novo assembly of genomes (Rhoads and Au 2015). We found that the number of SNPs per window was very similar between the two alignments based on Illumina and PacBio reads, including the amount of SNPs in the highly variable windows. Thirdly, we verified the haplotype patterns in 11 randomly selected high variation regions using a PCR assay. From these independent approaches, we conclude that the observed “outlier” regions are indeed regions with an exceptional extent of variation that are interspersed throughout the genome of *Z. tritici*. We note that a reference-based mapping approach failed to identify these regions due to the extent of divergence. Instead, the nonreference haplotypes appear as missing data in the read mapping assembly. This observation may explain why previous population genomic studies of *Z. tritici* have failed to identify these regions.

The HVRs often comprise of two or fewer haplotypes with high-sequence identity within haplotype groups (supplementary fig. S4, Supplementary Material online), a pattern that resembles the genomic pattern in the hybrid sister species *Z. pseudotritici* (Stukenbrock et al. 2012). To investigate the origin of this particular haplotype pattern in *Z. tritici*, we combined several approaches. We determined the topology of phylogenetic trees in 1 kb windows across an alignment of *Z. tritici* and its sister species and correlated this with another measure of introgression, Gmin (Geneva et al. 2015), and the distribution of the HVRs. Together these different estimates provide strong evidence for multiple regions with signatures of interspecific gene flow along the *Z. tritici* genome. These signatures are highly localized with an average length of 1,238 bp in *Z. tritici*, distributed throughout the genome, and comprise of >20% of the total genetic variation.

A smaller proportion of the HVRs do not exhibit a clear introgression signal. One reason that we fail to detect introgression in these windows may be that *Z. tritici* has hybridized with other species not included in our analyses. It is also possible that our approach is not sensitive to detect all signals of introgression; while HVRs can be detected in alignment blocks with very few sequences, the Gmin measure relies on a large number of sequences. Thus, by our strict criteria for calling signatures of introgression, it is possible that we underestimate the overall impact of this phenomenon in the *Z. tritici* genome.

Another process that may produce genealogies that are discordant with the species tree is ILS. Several methods have been developed to distinguish these two processes from each other, including the ABBA-BABA test (Durand et al. 2011). For the *Zymoseptoria* genus studied here, we find evidence for more complex hybridization histories with gene flow in different directions at different time points and involving multiple species. This more complex scenario of introgression prevent us from applying tests like the ABBA-BABA test. However, to distinguish between ILS and introgression, we analyzed the length distribution of the detected haplotypes in the HVRs (Racimo et al. 2015). Generation after generation recombination breaks up haplotypes into smaller fragments. Given the recombination rate and the speciation time between two lineages, it is possible to estimate the expected length of haplotypes along a given genome. In the *Zymoseptoria* genus, the recombination rate has been estimated previously from genomic data and determined from experimental crosses (Stukenbrock et al. 2011; Croll et al. 2015). Based on these measures and previously estimated split times between species, we find that the expected haplotype length is <1 kb, a value consistent with previous estimates (Stukenbrock *et al.* 2011). However, the observed HVRs showing discordant genealogies are considerably longer than expected in a model excluding introgression after speciation and without selection. Although ILS occurs throughout the genome of the *Zymoseptoria* species (Stukenbrock et al. 2011), it does not explain variation in the HVRs. Finally, another possible explanation for the maintenance of the long and highly divergent haplotypes is balancing selection (Charlesworth 2006). However, the observed patterns of nonmonophyly and low Gmin values comprise a relatively large proportion of the genomes of *Z. tritici* and *Z. ardabiliae*. We consider it unlikely that balancing selection can shape genetic variation in such a large proportion of the genomes.

Previous studies, including a recent study of the yeast species *Saccharomyces paradoxus*, demonstrated the emergence of new fungal populations via repeated events of hybridization (Eberlein et al. 2019). We speculate that recurrent hybridization likewise may be a driving force in shaping evolution in *Zymoseptoria* grass pathogens. In the *Z. tritici* genome, we most frequently identify *Z. pseudotritici* and *Z. brevis* as the source of the introgressed genomic regions and more rarely *Z. ardabiliae*. Interestingly, in > 50% of the windows, we could not identify one unique source species. Such pattern may reflect that these regions are subject to repeated introgressions by different species. In support of this hypothesis, we found a similar pattern of gene flow in the genome of the sister species *Z. ardabiliae*. The presence of the same genomic signatures in the two species suggests that the gene flow in the *Zymoseptoria* genus is not unidirectional and does not only occur from the wild species into the wheat infecting lineage *Z. tritici*. Rather, hybridization may occur more frequently among closely related *Zymoseptoria* species and may even be a mechanism for the exchange of adaptations between species. This scenario was previously shown to explain the mosaic genome structure observed most pronounced in *Z. pseudotritici* (Stukenbrock et al. 2012), but here also described in *Z. tritici* and *Z. ardabiliae*. A similar mosaic genome pattern has been reported previously in the biotrophic oomycete parasite, *Albugo candida*, in which 25% of the genome was found to be introgressed between different host-specific races (McMullan et al. 2015). In *A. candida*, introgression may be an important mechanism to facilitate host shifts as the introgression involves the exchange of host-specific effector genes. Likewise, it has been proposed that introgression has played an important role in the evolution of virulence traits in the fungal apple scab pathogen *Venturia inaequalis* (Leroy et al. 2016). It is possible that *Zymoseptoria* in its original habitat can similarly hybridize to facilitate host range expansion. To which extent introgression has impacted the virulence of the specialized pathogen has still yet to be assessed. Although the introgressed regions are not enriched with effector genes, they overlap with 18 predicted effector genes. This finding suggests that hybridization may be a mechanism whereby virulence determinants can be exchanged between *Zymoseptoria* species. Future experimental studies should elucidate the functional relevance of extensive allelic variation in these regions and the role of hybridization in the emergence of new host specificities.

We find an enrichment of TEs in the HVRs. It is possible to speculate that selective constraints will act more strongly on introgressed protein-coding sequences than on TEs, explaining the enrichment of these sequences in the HVRs. Hybridization may even act as a mechanism for TEs to be transferred between species. Comparative genome analyses of closely related *Drosophila* species recently provided evidence for the recurrent transfer of TEs between sympatric *Drosophila* species by hybridization (Hill and Betancourt 2018). The authors proposed that the transfer of TEs by hybridization contribute to the highly dynamic genome content in the fly genomes. For Zymoseptoria, we hypothesize that the high variability in TE content between and within closely related species likewise could be explained by hybridization.

The results of our study underline the prominent role of interspecific gene flow in the genome evolution of a prominent wheat pathogen. While the functional relevance of such exchanges of genetic material in this genus is unknown, it is a phenomenon that is widespread in the genus *Zymoseptoria*. Sister species of *Z. tritici* have been collected from a variety of wild grasses in Iran (Stukenbrock et al. 2006; Haueisen J, personal communication), and we hypothesize that hybridization facilitates host shifts and host range expansion. The frequent occurrence of hybridization among these plant pathogens raises questions about the way we define species as isolated entities, which has fundamental relevance to disease management and plant breeding. Future studies should focus on the mechanisms promoting or preventing hybridization as well as the relevance of hybridization in the evolution of virulence and host specificity. 

## Supplementary Material

Supplementary data are available at *Genome Biology and Evolution* online.

## Supplementary Material

evz224_Supplementary_Data
