# Supplementary material for: Interspecific Gene Exchange Introduces High Genetic Variability in Crop Pathogen
Source: Genome Biol Evol. 2019 Oct 11;11(11):3095–105. doi: 10.1093/gbe/evz224 (PMC6836716; doi:10.1093/gbe/evz224)
Supplement: evz224_Supplementary_Data [file evz224_supplementary_data.zip › Feurtey_GBE2019_SupportingInformationLegends.docx]

# Interspecific gene exchange introduces high genetic variability in crop pathogen

Alice Feurtey, Danielle M. Stevens, Wolfgang Stephan & Eva H. Stukenbrock

# Supplementary Material:

**Supplementary Tables**

**Table S1:** Overview of genome alignments used in this study.

**Table S2:** Summary statistics of genome alignments.

**Table S3:** List of primers used to verify HVRs.

**Table S4:** Summary of PCR assay to validate HVRs.

**Table S5:** Gene Ontology terms enriched in HVRs

**Supplementary Figures**

**Figure S1: Schematic representation of the pipeline used to produce the multi-genome alignments.** References for the different programs used are listed in the Materials and Methods.

**Figure S2: Genome synteny of *Zymoseptoria* genomes.** A) Circo plot of the *Z. tritici* IPOP323 reference genome (in yellow) and the PacBio assembly of *Z. triciti* Zt10 (in black). B) Circos plot of IPOP323 and the PacBio assembly of *Z. pseudotritici* Zp13 (dark green). C) Circos plot of IPOP323 and the Pac Bio assembly of *Z. brevis* Zb87 (in light green). D) Circo plot of IPOP323 and the Pac Bio assembly of *Z. ardabiliae* Za17 (in green). The connecting lines correspond to alignment blocks of more than 20kb in the pairwise alignment of the represented genomes.

**Figure S3: Overview of the synteny-based filter applied to the genome alignments.** The columns represent five different alignment scenarios, resulting from different filtering criteria (based on the thresholds indicated below the table). In all schematics, the reference chromosomes 1 and 2 are represented by green and yellow lines. The scaffold to filter and aligning to either reference chromosome is either in black when the portion of the scaffold passes filtering or in grey if it fails filtering. Each cell contains the percentage of the scaffold aligned to a given reference chromosome and the total aligned length to this chromosome. The bold letters indicate which number (of the length or the proportion) passed the filter and caused the fragment to be included in the filtered MGA. Scenarios 1 and 2 correspond to a scaffold aligning solely on one reference chromosome and thus kept in the MGA. In scenarios 3 to 5, the scaffold aligns partially to both of the reference chromosomes, which result in either filtering a portion of the scaffold or the whole scaffold.

**Figure S4: Introgression signature in genome alignment.** Exemplary alignment for three 100-bp loci on the chromosome 10 of *Z. tritici* (alignment block starting at position 838681). A) Conserved sequence with few randomly distributed SNPs until the beginning of the highly variable alignment block (B). This is followed by a considerably more conserved alignment with few SNPs (C).

**Figure S5: Comparison of variant calling in short and long read genome assemblies.** Comparison of the number of segregating sites per 1-kb window (sliding by 500 pb) using different variant detection methods but with the genome data. In the two plots, the x-axis represents the multiple genome alignment (MGA) created from the reference genome and two de novo assemblies based on PacBio sequencing reads. On the left, the y-axis represents the MGA created from the reference genome and two de novo assemblies based on Illumina reads. On the right, the y-axis represents mapping of Illumina reads on the reference genome followed by variant calling.
