# Supplementary material for: Interspecific Gene Exchange Introduces High Genetic Variability in Crop Pathogen
Source: Genome Biol Evol. 2019 Oct 11;11(11):3095–105. doi: 10.1093/gbe/evz224 (PMC6836716; doi:10.1093/gbe/evz224)
Supplement: evz224_Supplementary_Data [file evz224_supplementary_data.zip › S3_Figure_filtering.pdf]

	**Scenario 1**	**Scenario 2**	**Scenario 3**	**Scenario 4**	**Scenario 5**
**Schematic representation of the alignment**	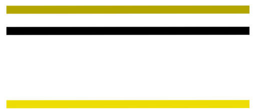	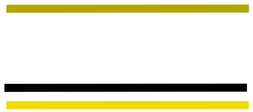	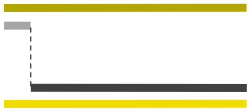	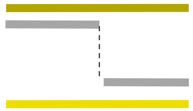	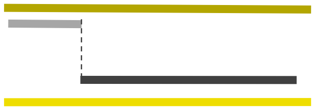
**Portion of the scaffold aligned on chromosome 1**	**100% | 20kb**<br>Kept	0% | 0kb<br>-	8% | 1.6kb<br>Filtered out	40% | 4kb<br>Filtered out	15% | 6kb<br>Filtered out
**Portion of the scaffold aligned on chromosome 2**	0% | 0kb<br>-	**100% | 20kb**<br>Kept	**92%** | 18.4kb<br>Kept	60% | 6kb<br>Filtered out	85% | **21kb**<br>Kept

Minimum aligned proportion = 90% ; Minimum aligned length = 20 kb

Reference chromosome 1

Reference chromosome 1

Aligned length of the scaffold passing filtering

Aligned length of the scaffold failing filtering
