# Supplementary figures and images for: Interspecific Gene Exchange Introduces High Genetic Variability in Crop Pathogen
Source: Genome Biol Evol. 2019 Oct 11;11(11):3095–105. doi: 10.1093/gbe/evz224 (PMC6836716; doi:10.1093/gbe/evz224)

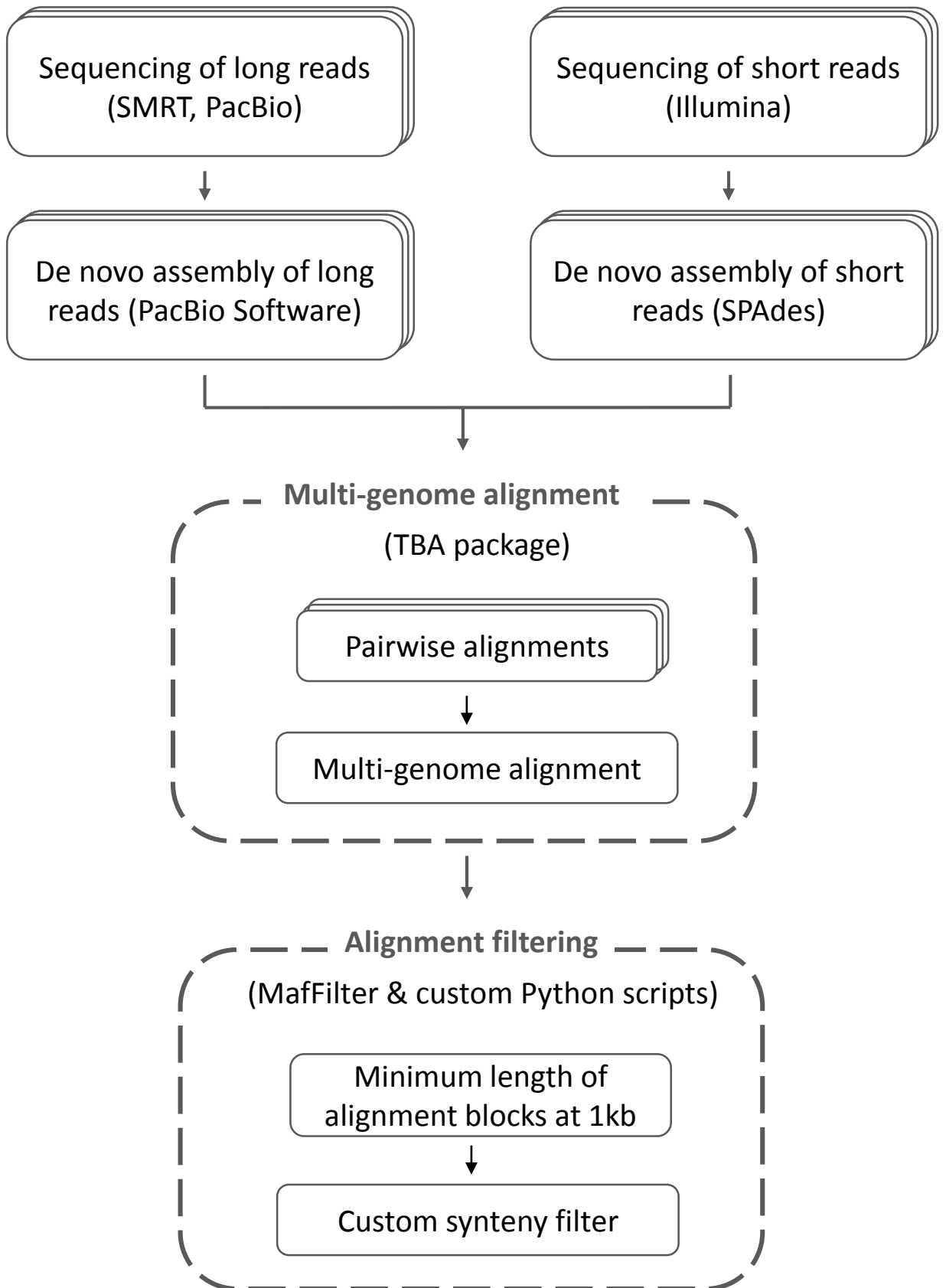

Supplement: evz224_Supplementary_Data [file evz224_supplementary_data.zip › S1_Figure_pipeline.pdf]

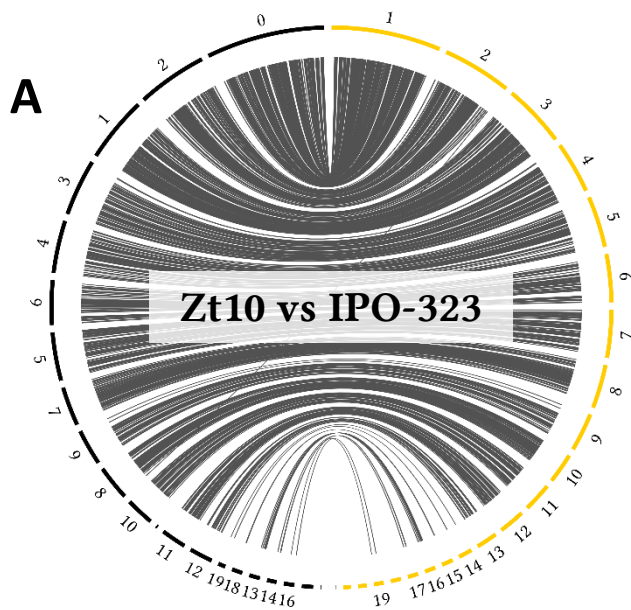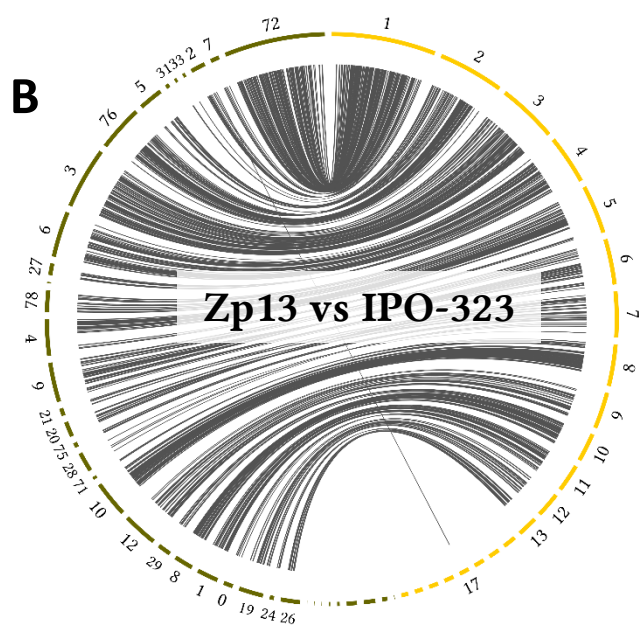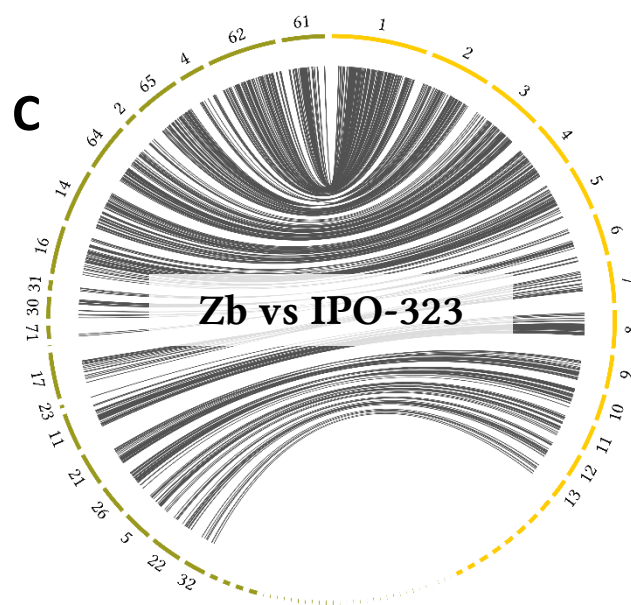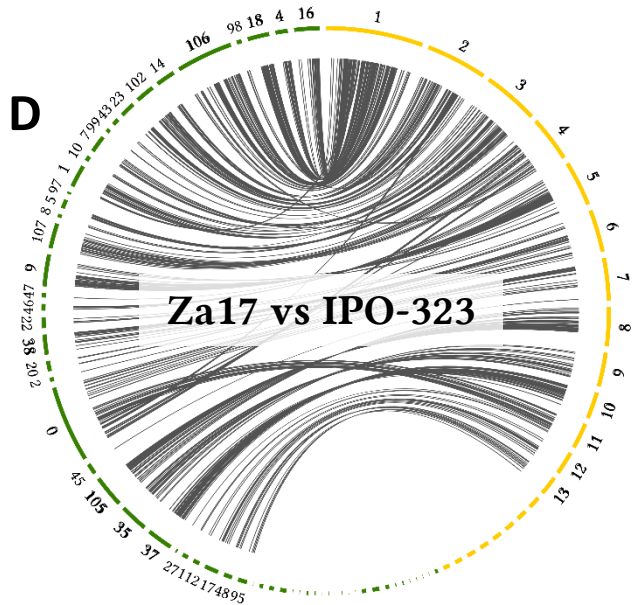

Supplement: evz224_Supplementary_Data [file evz224_supplementary_data.zip › S2_Fig_synteny_circos.pdf]
