# Supplementary material for: Interspecific Gene Exchange Introduces High Genetic Variability in Crop Pathogen
Source: Genome Biol Evol. 2019 Oct 11;11(11):3095–105. doi: 10.1093/gbe/evz224 (PMC6836716; doi:10.1093/gbe/evz224)
Supplement: evz224_Supplementary_Data [file S4_Figure_alignment.pdf › S4_Figure_alignment.pdf]

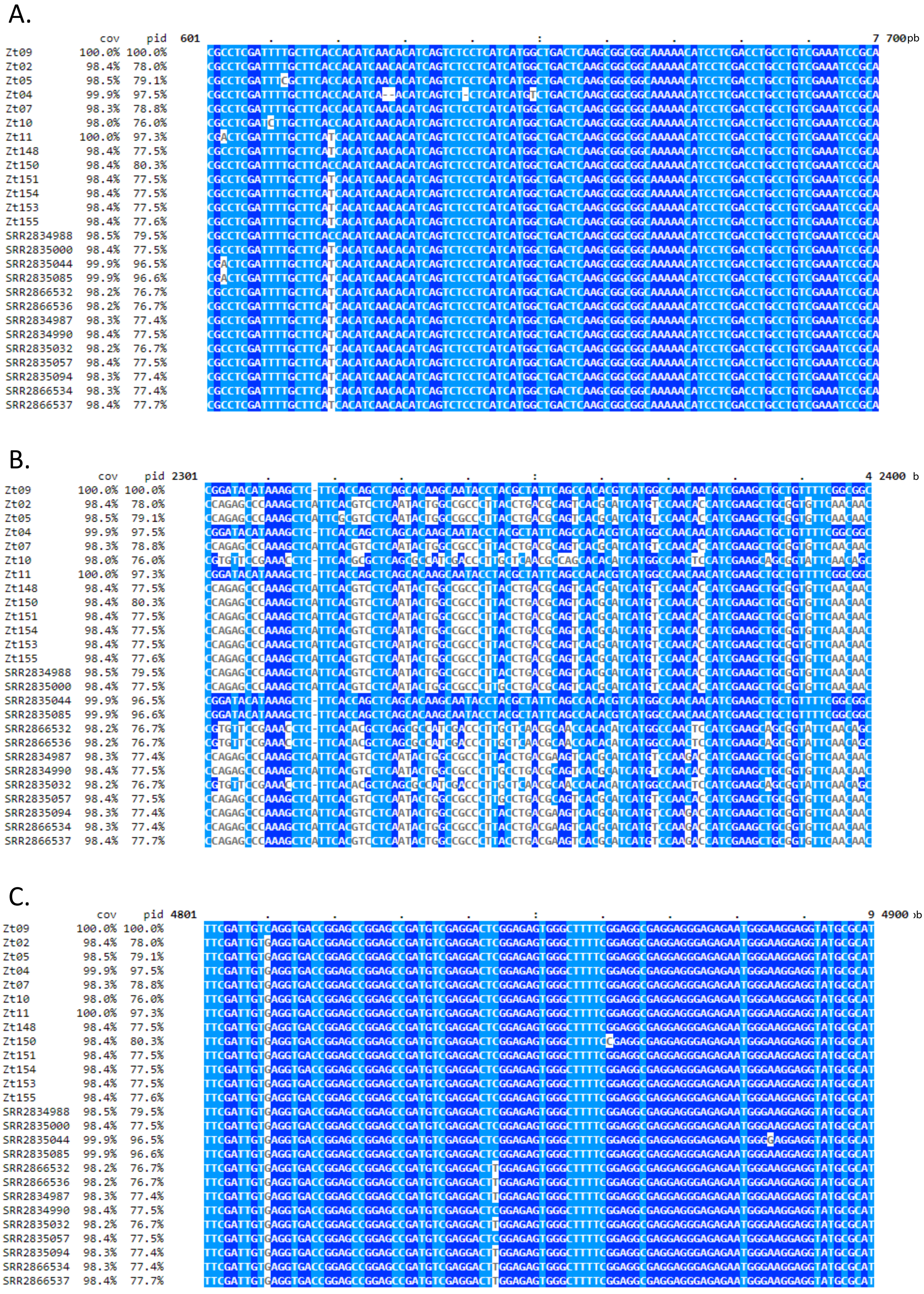

A.
cov pid 601 7 700pb
Zt09 100.0% 100.0%
Zt02 98.4% 78.0%
Zt05 98.5% 79.1%
Zt04 99.9% 97.5%
Zt07 98.3% 78.8%
Zt10 98.0% 76.0%
Zt11 100.0% 97.3%
Zt148 98.4% 77.5%
Zt150 98.4% 80.3%
Zt151 98.4% 77.5%
Zt154 98.4% 77.5%
Zt153 98.4% 77.5%
Zt155 98.4% 77.6%
SRR2834988 98.5% 79.5%
SRR2835000 98.4% 77.5%
SRR2835044 99.9% 96.5%
SRR2835085 99.9% 96.6%
SRR2866532 98.2% 76.7%
SRR2866536 98.2% 76.7%
SRR2834987 98.3% 77.4%
SRR2834990 98.4% 77.5%
SRR2835032 98.2% 76.7%
SRR2835057 98.4% 77.5%
SRR2835094 98.3% 77.4%
SRR2866534 98.3% 77.4%
SRR2866537 98.4% 77.7%
B.
cov pid 2301 4 2400 b
C.
cov pid 4801 9 4900
